# Vector Borne Infections in Italy: Results of the Integrated Surveillance System for West Nile Disease in 2013

**DOI:** 10.1155/2015/643439

**Published:** 2015-03-22

**Authors:** Christian Napoli, Simona Iannetti, Caterina Rizzo, Antonino Bella, Daria Di Sabatino, Rossana Bruno, Francesca Sauro, Vanessa Martini, Vincenzo Ugo Santucci, Silvia Declich, Paolo Calistri

**Affiliations:** ^1^Centro Nazionale di Epidemiologia, Sorveglianza e Promozione della Salute, Istituto Superiore di Sanità, Viale Regina Elena 299, 00161 Roma, Italy; ^2^Centro di Referenza Nazionale per l'Epidemiologia Veterinaria, la Programmazione, l'Informazione e l'Analisi del Rischio, Istituto Zooprofilattico Sperimentale dell'Abruzzo e del Molise “G. Caporale” Campo Boario, 64100 Teramo, Italy; ^3^Ministero della Salute, Via Ribotta 5, 00144 Roma, Italy

## Abstract

The epidemiology of West Nile disease (WND) is influenced by multiple ecological factors and, therefore, integrated surveillance systems are needed for early detecting the infection and activating consequent control actions. As different animal species have different importance in the maintenance and in the spread of the infection, a multispecies surveillance approach is required. An integrated and comprehensive surveillance system is in place in Italy aiming at early detecting the virus introduction, monitoring the possible infection spread, and implementing preventive measures for human health. This paper describes the integrated surveillance system for WND in Italy, which incorporates data from veterinary and human side in order to evaluate the burden of infection in animals and humans and provide the public health authorities at regional and national levels with the information needed for a fine tune response.

## 1. Introduction

The epidemiology of arboviral zoonoses is influenced by multiple ecological factors and, therefore, integrated and comprehensive surveillance systems are needed for early detecting the infection and activating consequent control actions. West Nile virus (WNV) is a* Flavivirus* belonging to the Japanese encephalitis antigenic complex of the family Flaviviridae. The genus* Flavivirus* also includes other arboviruses, such as St Louis encephalitis virus, Japanese encephalitis virus, Murray Valley virus, Usutu virus, and Kunjin virus. WNV is maintained in nature by birds and is transmitted primarily by the bite of infected mosquitoes acquiring the virus by feeding on infected birds [1]. Mosquitoes can also acquire the virus by transovarial transmission or by mating [2]. Migratory birds are strongly suspected to play a significant role in the introduction of WNV from endemic areas into new regions [3]. Humans, horses and other mammals are considered incidental dead-end hosts [1].

West Nile disease (WND) is a zoonosis. The infection in humans mainly occurs asymptomatically or with mild febrile illness [4]. Less than 1% of patients show severe neurological symptoms classifiable in three main syndromes: meningitis, encephalitis, and poliomyelitis (acute flaccid paralysis) [5]. In horses the disease is usually subclinical, although sometimes they may show neurological symptoms [1].

In the Western Hemisphere WNV was first detected in New York City in 1999 [6]. After that episode, the virus spread dramatically westward across the United States of America, southward into Central America and the Caribbean, and northward into Canada, resulting in the largest human epidemic of a neuroinvasive disease ever reported [7].

The first large human outbreak of WND in Europe was recorded in 1996 in Romania with 393 confirmed cases [8]. After that episode, the number of WND reported cases in horses and humans increased significantly. This apparent rise of reported cases is partly due to the improvement of both surveillance systems and diagnostic methods [3, 9, 10], and also to the introduction and rapid spread of WNV lineage 2 in Europe [11, 12]. In the last recent years, virus circulation was observed in different Mediterranean countries with an increasing number of human cases in the Eastern Europe [13]. Some countries (i.e., Greece, Spain, Russia, Israel, Hungary, and Romania) were affected by the virus circulation for several consecutive years, supporting the hypothesis of a possible local endemisation of the infection.

As various animal host species take part, with different epidemiological roles, to the transmission of WNV, a multispecies surveillance approach is required. Some European countries focused their surveillance program only on human population (i.e., Albania, Kosovo, Montenegro) [14], whereas others integrated these activities with general and/or targeted surveillance in equines (such as Croatia, Spain, Greece, Portugal, France, Romania, Cyprus, and Morocco) [14–16]. Moreover, in some European nations (i.e., in Italy, Greece, United Kingdom, Spain, Germany, Hungary, and Serbia) the surveillance on mosquito populations is added to that addressing humans and equines, with the aim of detecting the WNV early during the season [11, 12, 17]. In this regard, the highest percentage of mosquito pools tested positive for WNV was reached in Serbia in 2013 (5.5%) [12].

In Italy, the first outbreak of WND was identified in horses in Tuscany region during the late summer of 1998. Fourteen animals showed neurological disorders [18], but no cases of human encephalitis were reported [9]. Following this epidemic, a national veterinary surveillance plan was put in place in 2001 to identify the geographical areas at risk for reintroduction of the WNV infection. This surveillance plan was coordinated by the National Reference Centre for the study of Exotic Animal Diseases (CESME) and carried out in 15 Italian wetlands. It was based on entomological monitoring and periodical serological testing of sentinel chickens and equines [19, 20]. The surveillance system did not detect any relevant circulation of WNV in animals until 2008, when the virus was detected in mosquitoes, birds, equines, and humans in the area surrounding the Po river delta, involving eight provinces in three northern regions: Emilia Romagna, Veneto, and Lombardy [20–22].

Based on what happened during 2008, the national WNV veterinary surveillance system was revised and new activities were added, aiming at identifying as early as possible the virus circulation all over the country and implementing measures for the prevention and control of human infections [20].

In light of the increasing number of confirmed cases in animals and the circulation of the virus in a wider geographical area, a human surveillance plan for the West Nile neuroinvasive disease (WNND) was put in place in 2008 in Emilia Romagna and Veneto regions, which allowed to detect the first eight indigenous cases of WNND in humans [9].

In 2009, the results of the veterinary surveillance system showed the resurgence of infection mostly in the same geographical areas of previous year, but new foci were reported in central Italy, in Tuscany and Latium, rather far from the areas infected in 2008 [22]. Cases of human WNND increased to 18 in 2009 (nine cases in Emilia Romagna, seven in Veneto, and two in Lombardy regions), occurring in the same geographical areas where WNV circulation was detected in mosquitoes and animals (chickens and equine) [9].

In August 2010, new foci of infection were observed in Sicily and Molise regions, respectively, in southern and central Italy. These outbreaks confirmed the WNV ability of spreading to new areas, affecting new host populations [20, 23]. In 2011, WND outbreaks were confirmed in six regions: Sardinia, Sicily, Friuli Venetia Giulia, Veneto, Basilicata, and Calabria, where clinical cases in horses and neurological signs in birds were observed.

Between 2010 and 2011 seventeen new human cases of WNND were reported from three regions (Veneto, Friuli Venetia Giulia, and Sardinia) with a 23.5% case-fatality rate [9].

Following the geographical spread of  WNV westward, the Directorate General for Prevention of the Italian Ministry of Health (MoH) issued on the spring of 2010, a national plan for WNND human surveillance that integrated human and veterinary surveillance [24]. Since then, the national surveillance plan on imported and autochthonous human vector-borne disease (chikungunya, dengue and West Nile disease) was issued and revised annually [25–27].

In 2012 WNV continued to circulate in Italy causing infection in humans, horses, birds, and vectors in Sardinia, Friuli Venetia Giulia, Veneto, and Latium regions [10, 28, 29].

The main objective of this paper is to describe the components of the integrated surveillance system for WNV established in Italy with the aim of evaluating the burden of the disease in animals and humans and providing the Public Health local and national authorities with the needed information to fine tune response. To this aim, the existing data exchange flows between veterinary and human systems and the results of 2013 surveillance are presented.

## 2. Materials and Methods

### 2.1. WNV Circulation Surveillance Plan in Animals

**Figure 1:**
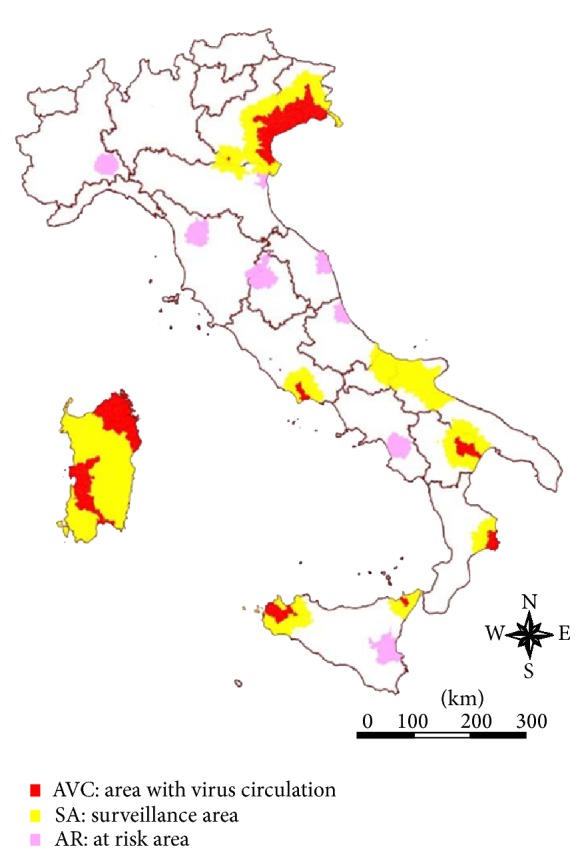
WND surveillance areas in 2013.

The Italian national veterinary authority annually revises the WNV veterinary surveillance plan in line with the observed epidemiological changes. The surveillance plan for 2013 delimits three different geographical areas (Figure 1):

- with virus circulation (AVC): the geographic areas affected by the circulation of WNV in the previous years (since 2008),
- surveillance zones (SZ): the territories surrounding the AVC with an extension of 20 km,
- at risk (AR): wetlands characterized by the presence of a significant number of migratory water fowls.

Target species of the veterinary surveillance activities include migratory and resident birds, horses, and poultry. The entomological surveillance is based on a certain number of mosquito collection sites placed in the three above mentioned areas for identifying possible WNV vector species and determining their abundance and spatiotemporal distributions [30].

Active bird surveillance is focussed on the following species: Magpie (*Pica pica*), Hooded Crow (*Corvus corone cornix*), and Jay (*Garrulus glandarius*), which are sampled and virologically tested in AVC. Serological testing of sentinel chickens and backyard poultry is foreseen as a possible alternative in case the planned activities on resident birds could be not carried out. Passive surveillance on birds mortality is carried out throughout the country, and target species include Blackbird (*Turdus meru*la), Starling (*Sturnus vulgaris*), Jackdaw (*Corvus monedula*), Magpie (*Pica pica*), Jay (*Garrulus glandarius*), Hooded Crow (*Corvus corone cornix*), and Collared dove (*Streptopelia decaocto*). In addition, any episode of abnormal or increased mortality in other wild birds must be reported to veterinary authorities.

Entomological surveillance aims at identifying the mosquito fauna, defining the composition of vector populations and the species responsible for WNV transmission in the enzootic and epizootic cycles of the disease, investigating their ability to overwinter.

Moreover, countrywide passive surveillance on neurological cases observed in equines is coupled with the serological survey performed in sentinel horses three times per year (in May, August, and September) in AR. In addition, when the viral circulation is detected in zones not previously affected by the infection, further activities are put in place to better identify the extent of the infection.

### 2.2. Veterinary WND Surveillance Information System

In 2008, the Department of Veterinary Public Health Nutrition and Food safety (VPH Department) of the MoH appointed the CESME to develop an information system collecting data on animal disease outbreaks, using standard procedures and templates for data input and output [31]. The new information system, called SIMAN, was developed to provide a tool for the management of epidemic emergencies, to collect and communicate outbreak data to the MoH, the European Commission and the World Organization of Animal Health (OIE) in compliance with current national and international legislation [32, 33]. SIMAN was firstly used by the veterinary services during the large WND epidemic occurred in 2008. The data reported to SIMAN allowed the veterinary services to have the full picture of outbreaks distribution and to plan further investigations [34].

Given the complex epidemiology of the disease and the multidisciplinary approach needed for its surveillance, an integrated and comprehensive system for the management of WND outbreaks and surveillance activities was established.

In particular, new tools were developed in SIMAN for

- the registration of sentinel chickens and equines into the National Database of livestock and holdings (BDN);
- recording and managing the laboratory results;
- publishing weekly and daily reports describing the outcomes of the surveillance activities.

A web-based geographic information system (WebGIS) was also developed for displaying thematic maps and to help the veterinary services to explore the area surrounding the outbreak, to create buffers around the reported cases, and to download the list of equine farms placed within the buffers. An automatic procedure extracting every night all data inconsistencies and errors assured the necessary quality checks. In case of errors, an automatic alert email is sent to the veterinary services asking for data verification and correction.

### 2.3. WNND Surveillance Plan in Humans

The national plan for human surveillance defines as “affected areas” all the provinces (secondary administrative units) where laboratory-confirmed WNV infections in animals, vectors, or humans have been notified in the previous year or during the current surveillance period (between 15 June and 30 November, as considered the period with the highest vector activity). The identification of an affected area immediately triggers the “surveillance area” that represents the regional territory of the affected area [27].

In the affected area, local health authorities have to implement an active surveillance system for WNND in workers employed in the farms where equine cases have been identified and in individuals living or working in the surrounding area (province). Moreover, the measures for vector control have to be implemented immediately. At the same time, passive surveillance on human neurological cases has to be set up in the surveillance area, requesting physicians to report all probable and confirmed WNND cases using a modified European case definition [35]: a patient with fever ≥38.5°C and neurological symptoms (encephalitis, meningitis or Guillain-Barré syndrome or acute flaccid paralysis) and at least one of the following laboratory criteria:

- for probable case: anti-WNV specific antibody response in blood; Polimerase Chain Reaction (PCR) positive in urine;
- for confirmed case: viral isolation in blood or cerebrospinal fluid (CSF); anti-WN IgM positive in CSF; PCR positive in blood or CSF; confirmed presence of anti-WN antibodies in blood by neutralization test.

The list of regional reference laboratories is also provided in the WNND national surveillance plan. When a probable case is reported, the regional reference laboratory has to proceed for confirmation using one of the above reported laboratory methods. In case the neutralisation test is not available at the regional laboratory, patient's sera are sent to the National Reference Laboratory at Istituto Superiore di Sanità (ISS) for further confirmatory tests.

When WNND human cases are confirmed, immediate WNV nucleic acid amplification test (NAAT) screening of all blood and haematopoietic stem cells donations must be ordered in the affected areas. Additional screening of solid organ donations in surveillance areas (regions) are also introduced [36]. At the national level, all blood, tissue and solid organ donors who travelled to an affected area have to be temporarily deferred for 28 days starting with the day they left the affected area [37].

### 2.4. Case Reporting System of Human WNND

All human cases are notified by regional authorities to the MoH and to the Italian National Centre for Epidemiology, Surveillance and Health Promotion (CNESPS-ISS) using a specific password-protected web-based system (http://www.simi.iss.it/inserimento_dati.htm), which permits to report probable and confirmed cases, adding available epidemiological, clinical and laboratory information. The database is accessible also for the National Blood Centre and to the National Transplant Network, which implements precautionary measures on blood donation and transplant activities on the basis of data on WNND human cases.

In addition to the activities foreseen by the national WNND surveillance, an enhanced regional surveillance for WNV fever (WNF) was established in Veneto, Emilia Romagna, and Lombardy regions. The case definition for WNF was the following: a person showing fever ≥38.5°C (or history of fever in the last 24 h) for a period no longer than seven days, from 15 July to 30 November, with no recent history of travels to tropical countries and absence of other comorbidities accounting for the febrile illness.

All WNF confirmed cases are notified by the regional authorities to the MoH and to the CNESPS-ISS through the web-based system.

### 2.5. The Surveillance Systems Integration

During the vector activity period, a data exchange protocol is in place between SIMAN and the CNESPS-ISS to jointly define and update the map of the affected areas (provinces). The identification of new ACV and AE following the veterinary activities immediately triggers the establishment of the “affected areas” and the “surveillance areas” as foreseen by the human WNND surveillance plan. When an outbreak is confirmed in SIMAN or a laboratory result confirms WNV circulation in a given territory, measures for the prevention and control of the infection in humans are immediately applied in the affected areas. Veterinary and human surveillances are, therefore, linked each other and work as a chain reaction.

Together with the animal and entomological monitoring, the surveillance of WNND human cases allows to detect the virus circulation in a given geographical area and to obtain an estimation of its magnitude through the systematic detection of emerging clinical cases.

**Figure 2:**
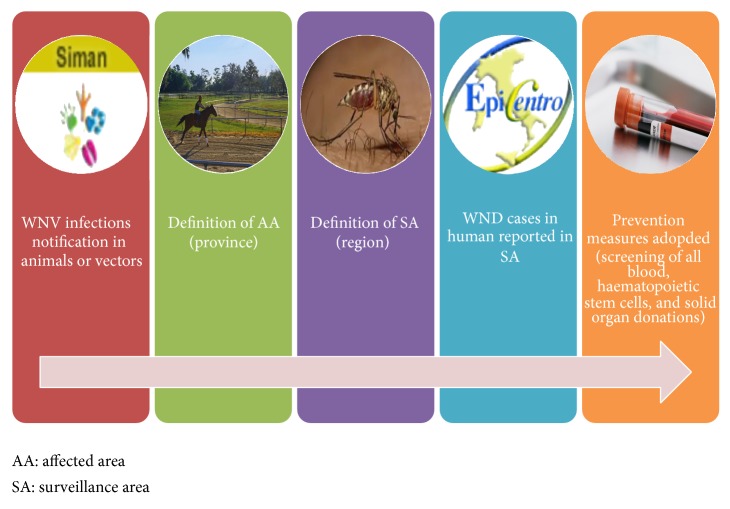
The web-based integrated surveillance system.

The data flow in the web-based integrated surveillance system is shown in Figure 2.

## 3. Results

### 3.1. Animal and Entomological Surveillance

In 2013, the CESME confirmed 50 new cases of WND in equines, 12 of which characterized by clinical signs, in Veneto, Lombardy, Emilia Romagna, Calabria, Sardinia, and Sicily regions; 146 mosquito pools and 79 birds in Veneto, Lombardy, Emilia Romagna, and Sardinia regions tested positive for WNV PCR. The affected areas identified by the veterinary monitoring activities were published on line (http://sorveglianza.izs.it/emergenze/west_nile/emergenze_en.html).

### 3.2. WNND Human Surveillance

**Figure 3:**
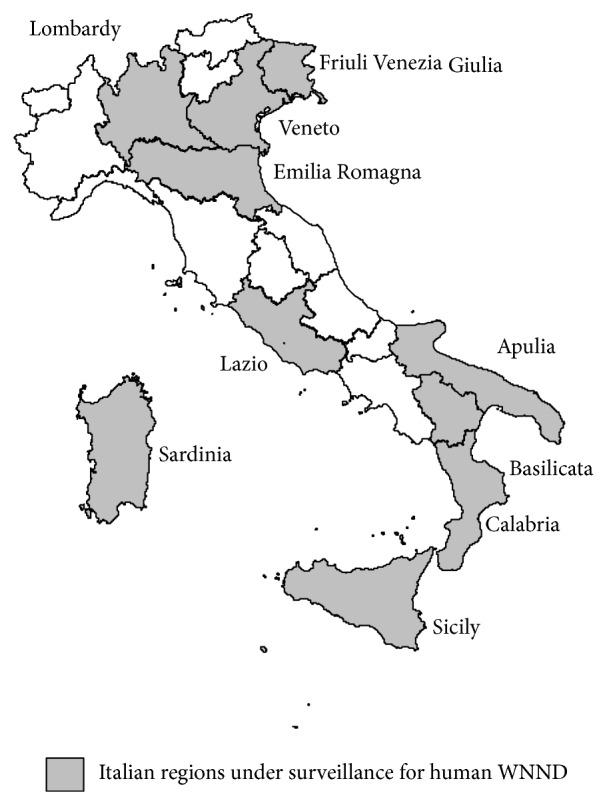
Regions under surveillance for human WNND, Italy 2013.

From 15 June to 30 November 2013, 44 autochthonous human cases of WNND were confirmed. The majority of patients were male (61.3%) with a median age of 73 years (range: 42–89 years). The onset of cases ranged between 21 July and 21 September: 75% had the symptoms onset in August, which represented the peak month in 2013 (Figure 3). None of the cases travelled abroad during the incubation period.

The distribution of WNND confirmed cases by age and region/province of exposure is showed in Table 1: the majority of WNND cases in 2013 were reported from Emilia Romagna (20 cases) followed by Veneto (13 cases), Lombardy (10 cases), and Apulia (1 case).

The majority of cases reported symptoms of encephalitis (70.5%), followed by meningitis (38.6%), polyradiculoneuritis (9.1%), and other neurological symptoms (18.2%). None of the patients had history of vaccination against other arboviruses. Seven cases died, corresponding to a 16.3% case fatality rate.

From 15 June to 30 November, 34 confirmed cases of WNF were also reported to the MoH and CNSPS-ISS by the public health authorities of Emilia Romagna (20 cases), Veneto (13 cases), and Lombardy (1 case) regions.

During the surveillance period the CNESPS-ISS published a weekly bulletin available in electronic format on the website of the ISS (http://www.epicentro.iss.it).

### 3.3. Outcomes from the Integrated Surveillance System

Data collected by both the veterinary and human surveillance systems in the previous year (2012) allowed to identify 9 regions (surveillance areas) in which the WNV human surveillance had to be performed in 2013 (Basilicata, Latium, Friuli Venetia Giulia, Sardinia, Veneto, Emilia Romagna, Lombardy, Calabria, and Sicily). Moreover, considering the geographical characteristics of Basilicata, also its neighbouring region (Apulia), although not directly affected, was included in the surveillance areas as well. Figure 3 shows the ten Italian Regions under surveillance.

During 2013 the veterinary surveillance activities confirmed the circulation of WNV in six regions (Sardinia, Veneto, Emilia Romagna, Lombardy, Calabria, and Sicily), of which three reported human cases (Veneto, Emilia Romagna, and Lombardy). One human case was reported also in Apulia, although no animals and vectors tested positive for WNV.

## 4. Discussion

The existence of surveillance systems able to represent an early warning tool is pivotal for preventing the spread of infectious diseases. The rapidity of the application of appropriate control measures after the detection of an emerging infectious/disease is crucial for the success of any intervention.

Between the first occurrence of WND in Italy in 1998 and its reemergence in 2008, WND was considered an exotic disease for the Italian territory and the main objective of the surveillance activities in place at that time was to evaluate the possible reintroduction of the virus. In this context, the data collected by the national system for the notification of animal diseases (SIMAN) were useful for a rapid epidemiological evaluation, to define areas at risk for human transmission and to facilitate the implementation of effective and prompt control measures.

**Figure 4:**
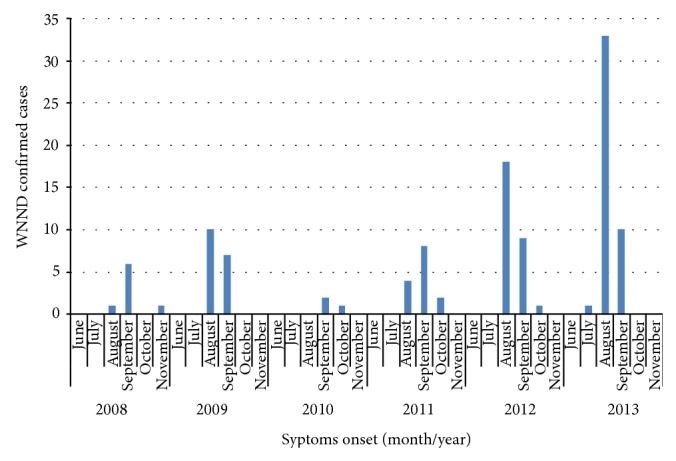
WNND confirmed cases by date of symptoms onset, Italy 2008–2013.

In Italy the first WNND human cases were detected in 2008, when a human surveillance system was implemented in the areas where the WNV circulation was demonstrated among animals and vectors. Since then, human cases of WNND have been reported every year in Italy, with a pick of incidence in 2013 (44 confirmed cases). As shown in Figure 4, the number of reported WNND human cases has been recently increased, due to the greater attention to the disease by the national authorities, and thanks to a better integration of human and veterinary surveillance.

In fact, an early warning system for WNV detection, based on animal and entomological surveillance, can provide the basis for targeted public health interventions and risk communication activities, aiming at reducing the risk of human infection. Since the first occurrence of the virus the multispecies surveillance plan in place in Italy was capable of confirming the WNV ability of spreading to new geographical areas and infecting different host populations. Veterinary surveillance activities, therefore, were particularly useful to assess and monitor the evolution of the epidemiological situation, providing the public health authorities with precious and timely information on where and when WND prevention and control actions in humans had to be put in place. The existence of an effective double way communication system between veterinarian and human health authorities ensured a prompt implementation of the preventive measures and a more accurate assessment of the epidemiological situation of the disease as well as a more precise estimation of the extension of the infection.

Well-conducted veterinary surveillance program allowed to identify the territories at major risk for WNV circulation and to set up human surveillance activities in these geographical areas. It is noteworthy that some regions, where WNV circulation was demonstrated in animal and vectors, did not reported any human case. On the contrary, in Apulia region, one autochthonous human case of WNND was confirmed in 2013, although no WND cases in horses or in other animal species were reported, although the detection three years before of a sporadic virus circulation in poultry farms in a bordering territory highlighted the suitability of that area for virus transmission. These findings confirm the crucial role of an integrated human, animal and vector surveillance in order to timely set up preventive measures. In this context, the veterinary surveillance carried out in sentinel animals, bird and vector populations can play a crucial role for foreseeing human transmission, even considering the limits of sensitivity of a surveillance system for vector-borne diseases.

Entomological surveillance is also a central aspect, allowing at early detecting the circulation of the virus [38–40]. In some provinces of northern Italy, the detection of WNV circulation through entomological surveillance was as early as July, largely more in advance than human cases occurrence [38].

With regard to circulating viruses, in the last three years WNV lineage 2 was detected in several Italian foci, apparently showing an extension of its spread and a more important contribution played by this lineage in the overall epidemiological situation in Italy. In addition, the co-circulation of lineages 1 and 2 in the same area [22] may create the favourable conditions for possible changes in the virulence of the viral strains, potentially leading to unexpected and adverse consequences.

## 5. Conclusions

The integrated human and veterinary information systems provide the Competent Authority with a large amount of data and information on WNV circulation, thus, allowing the evaluation of planned actions and, if needed, their improvement or revision. In 2013, the integrated human, entomological, and animal surveillance system was able to monitor the spread of WNV and supported the application of control measures for blood transfusions and organ donations, preventing the transmission of the disease among human population.

In conclusion, the Italian experience represents a good example of collaboration among different sectors of public health (human, veterinary, entomologists, and blood and organ donation authorities) in a “one health” perspective [41]. Vector borne diseases, in fact, need a multidisciplinary and integrated approach, that is more effective to assure animal and human health, as well as the environment protection. In case of zoonoses, such as WND, this approach is of paramount importance for a better and holistic understanding of the prevention of the diseases and the maintenance of both human and animal health.

## Figures and Tables

**Table 1:** WNND confirmed cases by region/province of exposure and age group, Italy 2013.

Region/province	Age					Total
	≤14	15–44	45–64	65–74	≥75	
Lombardy						
*Cremona *					1	1
*Mantua *			2	2	2	6
*Lodi *			1			1
*Brescia *					2	2
Apulia						
*Foggia *				1		1
Veneto						
*Rovigo *				1	4	5
*Treviso *			3		1	4
*Venice *					2	2
*Padua *					1	1
*Verona *					1	1
Emilia-Romagna						
*Bologna *					1	1
*Ferrara *		1	1	1	2	5
*Modena *			3	1	3	7
*Parma *					1	1
*Reggio Emilia *				1	5	6

Total	0	1	10	7	26	44
